# Supplementary material for: Genome-Wide Diversity of MADS-Box Genes in Bread Wheat is Associated with its Rapid Global Adaptability
Source: Front Genet. 2022 Jan 17;12:818880. doi: 10.3389/fgene.2021.818880 (PMC8801776; doi:10.3389/fgene.2021.818880)
Supplement: Supplementary file 1 [file DataSheet3.doc]

**Genome-Wide Diversity of MADS-Box Genes in Bread Wheat is Associated with its Rapid Global Adaptability**

**Frontiers in Genetics**

Qasim Raza^*^, Awais Riaz, Rana Muhammad Atif, Babar Hussain, Iqrar Ahmad Rana, Zulfiqar Ali, Hikmet Budak and Ibrahim A. Alaraidh

^*^ Correspondence: [qasimnazami@gmail.com](mailto:qasimnazami@gmail.com)

DOI:[10.3389/fgene.2021.818880](https://doi.org/10.3389/fgene.2021.818880)

**Supplementary references in Table S4:**

1. Ciaffi, M.; Paolacci, A.R.; D’Aloisio, E.; Tanzarella, O.A.; Porceddu, E. Identification and characterization of gene sequences expressed in wheat spikelets at the heading stage. *Gene* **2005**, doi:10.1016/j.gene.2004.11.004.

2. Clavijo, B.J.; Venturini, L.; Schudoma, C.; Accinelli, G.G.; Kaithakottil, G.; Wright, J.; Borrill, P.; Kettleborough, G.; Heavens, D.; Chapman, H.; et al. An improved assembly and annotation of the allohexaploid wheat genome identifies complete families of agronomic genes and provides genomic evidence for chromosomal translocations. *Genome Res.* **2017**, doi:10.1101/gr.217117.116.

3. Fu, D.; Szűcs, P.; Yan, L.; Helguera, M.; Skinner, J.S.; Von Zitzewitz, J.; Hayes, P.M.; Dubcovsky, J. Large deletions within the first intron in VRN-1 are associated with spring growth habit in barley and wheat. *Mol. Genet. Genomics* **2005**, doi:10.1007/s00438-004-1095-4.

4. Hama, E.; Takumi, S.; Ogihara, Y.; Murai, K. Pistillody is caused by alterations to the class-B MADS-box gene expression pattern in alloplasmic wheats. *Planta* **2004**, doi:10.1007/s00425-003-1157-6.

5. Hirabayashi, C.; Murai, K. Class C MADS-box gene AGAMOUS was duplicated in the wheat genome. *Wheat Inf. Serv* **2009**, *107*, 13–16.

6. IWGSC. Shifting the limits in wheat research and breeding using a fully annotated reference genome. *Science (80-. ).* **2018**, *361*, doi:10.1126/science.aar7191.

7. Kane, N.A.; Danyluk, J.; Tardif, G.; Ouellet, F.; Laliberté, J.F.; Limin, A.E.; Fowler, D.B.; Sarhan, F. TaVRT-2, a member of the StMADS-11 clade of flowering repressors, is regulated by vernalization and photoperiod in wheat. *Plant Physiol.* **2005**, doi:10.1104/pp.105.061762.

8. Ma, J.; Yang, Y.; Luo, W.; Yang, C.; Ding, P.; Liu, Y.; Qiao, L.; Chang, Z.; Geng, H.; Wang, P.; et al. Genome-wide identification and analysis of the MADS-box gene family in bread wheat (Triticum aestivum L.). *PLoS One* **2017**, doi:10.1371/journal.pone.0181443.

9. Meguro, A.; Takumi, S.; Ogihara, Y.; Murai, K. WAG, a wheat AGAMOUS homolog, is associated with development of pistil-like stamens in alloplasmic wheats. *Sex. Plant Reprod.* **2003**, doi:10.1007/s00497-002-0158-0.

10. Mizumoto, K.; Hatano, H.; Hirabayashi, C.; Murai, K.; Takumi, S. Altered expression of wheat AINTEGUMENTA homolog, WANT-1, in pistil and pistil-like transformed stamen of an alloplasmic line with Aegilops crassa cytoplasm. *Dev. Genes Evol.* **2009**, doi:10.1007/s00427-009-0275-y.

11. Paolacci, A.R.; Tanzarella, O.A.; Porceddu, E.; Varotto, S.; Ciaffi, M. Molecular and phylogenetic analysis of MADS-box genes of MIKC type and chromosome location of SEP-like genes in wheat (Triticum aestivum L.). *Mol. Genet. Genomics* **2007**, doi:10.1007/s00438-007-0285-2.

12. Ruelens, P.; De Maagd, R.A.; Proost, S.; Theißen, G.; Geuten, K.; Kaufmann, K. FLOWERING LOCUS C in monocots and the tandem origin of angiosperm-specific MADS-box genes. *Nat. Commun.* **2013**, doi:10.1038/ncomms3280.

13. Schilling, S.; Kennedy, A.; Pan, S.; Jermiin, L.S.; Melzer, R. Genome-wide analysis of MIKC-type MADS-box genes in wheat: pervasive duplications, functional conservation and putative neofunctionalization. *New Phytol.* **2020**, *225*, 511–529, doi:10.1111/nph.16122.

14. Shi, S. Ya; Zhang, F. Fei; Gao, S.; Xiao, K. Expression pattern and function analyses of the MADS thranscription factor genes in wheat (Triticum aestivum L.) under phosphorus-starvation condition. *J. Integr. Agric.* **2016**, doi:10.1016/S2095-3119(15)61167-4.

15. Shitsukawa, N.; Tahira, C.; Kassai, K.I.; Hirabayashi, C.; Shimizu, T.; Takumi, S.; Mochida, K.; Kawaura, K.; Ogihara, Y.; Muraia, K. Genetic and epigenetic alteration among three homoeologous genes of a class E MADS box gene in hexaploid wheat. *Plant Cell* **2007**, doi:10.1105/tpc.107.051813.

16. Wang, Q.H.; Yang, Z.J.; Wei, S.H.; Jiang, Z.Y.; Yang, Y.F.; Hu, Z.S.; Sun, Q.X.; Peng, Z.S. Molecular cloning, characterization and expression analysis of WAG-1 in the pistillody line of common wheat. *Genet. Mol. Res.* **2015**, doi:10.4238/2015.October.16.12.

17. Xiang, Y.Z.; Zhi, J.C.; Xian, S.Z. Overexpression of TaMADS1, a SEPALLATA-like gene in wheat, causes early flowering and the abnormal development of floral organs in Arabidopsis. *Planta* **2006**, doi:10.1007/s00425-005-0123-x.

18. Yamada, K.; Saraike, T.; Shitsukawa, N.; Hirabayashi, C.; Takumi, S.; Murai, K. Class D and Bsister MADS-box genes are associated with ectopic ovule formation in the pistil-like stamens of alloplasmic wheat (Triticum aestivum L.). *Plant Mol. Biol.* **2009**, doi:10.1007/s11103-009-9504-z.

19. Zhao, T.; Ni, Z.; Dai, Y.; Yao, Y.; Nie, X.; Sun, Q. Characterization and expression of 42 MADS-box genes in wheat (Triticum aestivum L.). *Mol. Genet. Genomics* **2006**, doi:10.1007/s00438-006-0147-3.
