# Supplementary material for: Genome-Wide Diversity of MADS-Box Genes in Bread Wheat is Associated with its Rapid Global Adaptability
Source: Front Genet. 2022 Jan 17;12:818880. doi: 10.3389/fgene.2021.818880 (PMC8801776; doi:10.3389/fgene.2021.818880)
Supplement: Supplementary file 2 [file DataSheet1.docx]

**Genome-Wide Diversity of MADS-Box Genes in Bread Wheat is Associated with its Rapid Global Adaptability**

**Frontiers in Genetics**

Qasim Raza^*^, Awais Riaz, Rana Muhammad Atif, Babar Hussain, Iqrar Ahmad Rana, Zulfiqar Ali, Hikmet Budak and Ibrahim A. Alaraidh

^*^ Correspondence: [qasimnazami@gmail.com](mailto:qasimnazami@gmail.com)

DOI:[10.3389/fgene.2021.818880](https://doi.org/10.3389/fgene.2021.818880)

**Figure S1** Comparative phylogenetic tree between *Arabidopsis* and wheat MADS-box genes. Arabidopsis and wheat MADS-box proteins were MAFFT aligned using only MADS domain (L-INS-i algorithm), maximum likelihood phylogenies inferred using I_Q_-T_REE_ software and final tree visualized with MEGA7. M- and MIKC-type genes were highlighted with green and red colour nodes, respectively.

**Figure S2** Comparative phylogenetic tree between rice and wheat MADS-box genes. Rice and wheat MADS-box proteins were MAFFT aligned using only MADS domain (L-INS-i algorithm), maximum likelihood phylogenies inferred using I_Q_-T_REE_ software and final tree visualized with MEGA7. M- and MIKC-type genes were highlighted with green and red colour nodes, respectively.


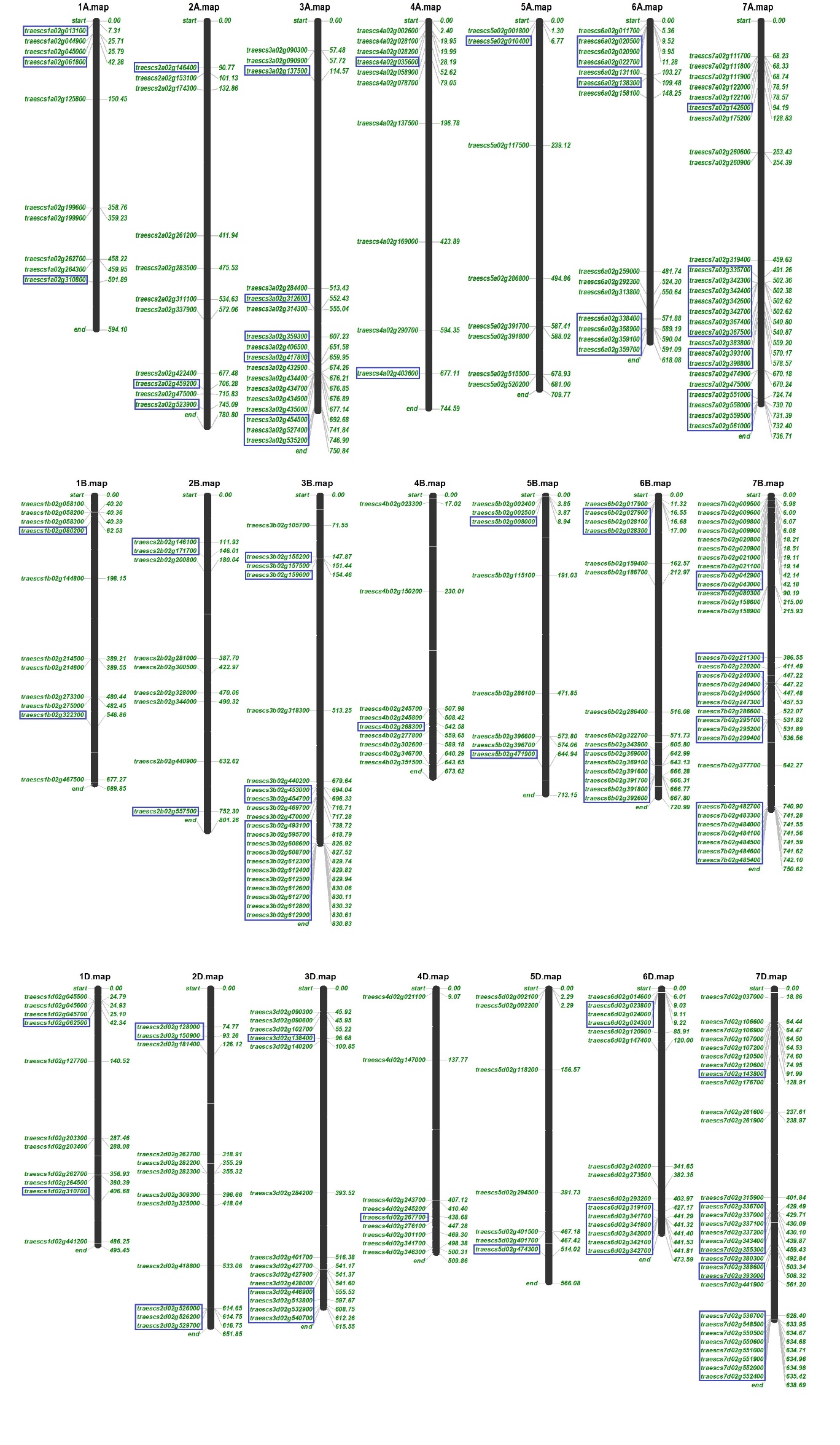


**Figure S3** Chromosomal mapping of wheat MADS-box genes. Solid black vertical bars represent individual chromosomal lengths (Mbp). Gene names and their initial physical positions are drawn on the left and right side of the respective chromosomes, respectively. M-type genes are encircled with blue boxes.


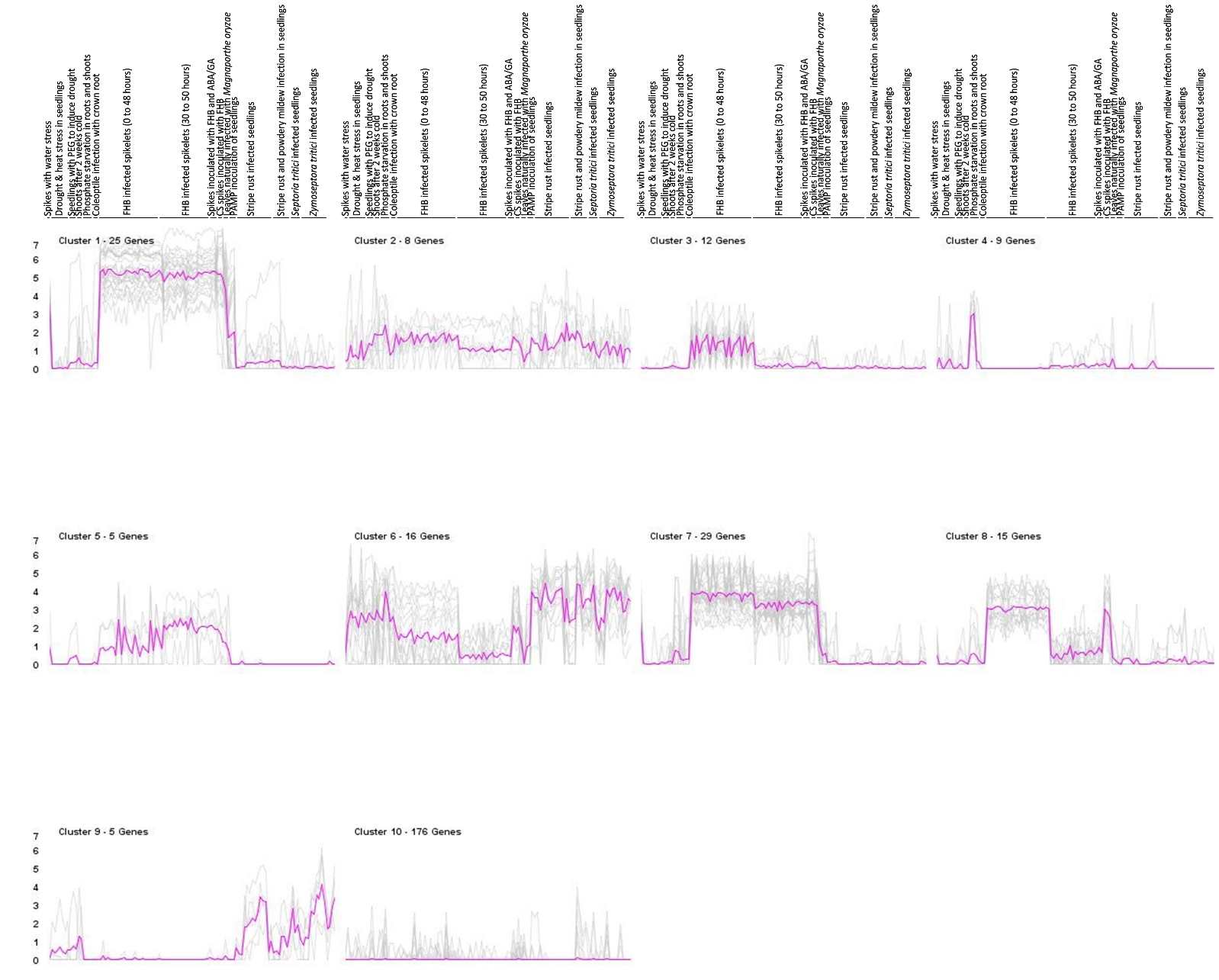


**Figure S4** Expression patterns based clustering of wheat MADS-box genes. Expression patterns based clustering of genes was achieved by following the K-means clustering method (K = 10, iterations = 1000, runs = 5) with Genesis software (**Table S8**).
